# Developmental Trends and Research Hotspots in Bronchoscopy Anesthesia: A Bibliometric Study

**DOI:** 10.3389/fmed.2022.837389

**Published:** 2022-06-30

**Authors:** Keting Min, Yutong Wu, Sheng Wang, Hao Yang, Huimin Deng, Juan Wei, Xiaowei Zhang, Huanping Zhou, Wanli Zhu, Yang Gu, Xuan Shi, Xin Lv

**Affiliations:** ^1^Graduate School, Wannan Medical College, Wuhu, China; ^2^Department of Anesthesiology, Shanghai Pulmonary Hospital, Tongji University School of Medicine, Shanghai, China; ^3^Department of Anesthesiology, Yangpu Hospital Affiliated to Tongji University, Shanghai, China

**Keywords:** VOSviewer, CiteSpace, bibliometric, bronchoscopy, anesthesia

## Abstract

**Background:**

This study discusses the developmental trends and research hotspots in bronchoscopy anesthesia in the past six decades.

**Methods:**

The original and review articles published from 1975 to June 2021 related to bronchoscopy anesthesia were retrieved from the Web of Science Core Collection (WoSCC). Three different scientometric tools (CiteSpace, VOSviewer, and Bibliometrix) were used for this comprehensive analysis.

**Results:**

There was a substantial increase in the research on bronchoscopy anesthesia in recent years. A total of 1,270 publications were retrieved up to June 25, 2021. Original research articles were 1,152, and reviews were 118, including 182 randomized controlled trials (RCTs). These publications were cited a total of 25,504 times, with a mean of 20.08 citations per publication. The US had the largest number of publications (27.6%) and the highest H-index of 44. The sum of publications from China ranked second (11.5%), with an H-index of 17. Keyword co-occurrence and references co-citation visual analysis showed that the use of sedatives such as dexmedetomidine in the process of bronchoscopy diagnosis and treatment was gradually increasing, indicating that bronchoscopy anesthesia was further progressing toward safety and comfort.

**Conclusion:**

Based on a bibliometric analysis of the publications over the past decades, a comprehensive analysis indicated that the research of bronchoscopy anesthesia is in a period of rapid development and demonstrated the improvement of medical instruments and surgical options that have significantly contributed to the field of bronchoscopy anesthesia. The data would provide future directions for clinicians and researchers in relation to bronchoscopy anesthesia.

## Background

Bronchoscopy is the main means for the examination, diagnosis, and treatment of various airway diseases and conditions involving the tracheobronchial tree (1–3). Fiberoptic bronchoscopy (FB) examination is an invasive examination, causing more harmful irritations such as severe reflux and aspiration in patients than gastroscopy. Pain and fear make some patients reject the FB procedure and not cooperate (4). However, the application of anesthesia can relieve the pain and nervousness in patients and promote the wide use of painless FB. Therefore, anesthesia is also an important link in the diagnosis and treatment process using bronchoscopy (5). Not only the complexity and risk of the operation but also the safety, comfort, and feelings of the patient need to be factored in when planning the anesthesia.

Bibliometrics is a quantitative and qualitative analysis method that uses mathematical and statistical tools to measure the interrelationships and impacts of publications within a given area of research (6, 7). This method can assess the publications and developmental trends in a scientific field and reveal key research directions by analyzing databases and characteristics of the publications. Particularly, bibliometric analysis can be used to summarize developmental trends and research hotspots of various diseases, such as acute lung injury, sacral fracture surgery, and pain treatment (8–10). However, there are few quantitative studies that analyze the research on bronchoscopy anesthesia. In this study, we used bibliometrics to analyze the development, trends, and hotspots of research on bronchoscopy anesthesia, hoping that the results could provide clinical anesthesiologists and related researchers with useful information in this field.

## Materials and Methods

### Data Sources and Retrieval Strategies

**Figure 1:**
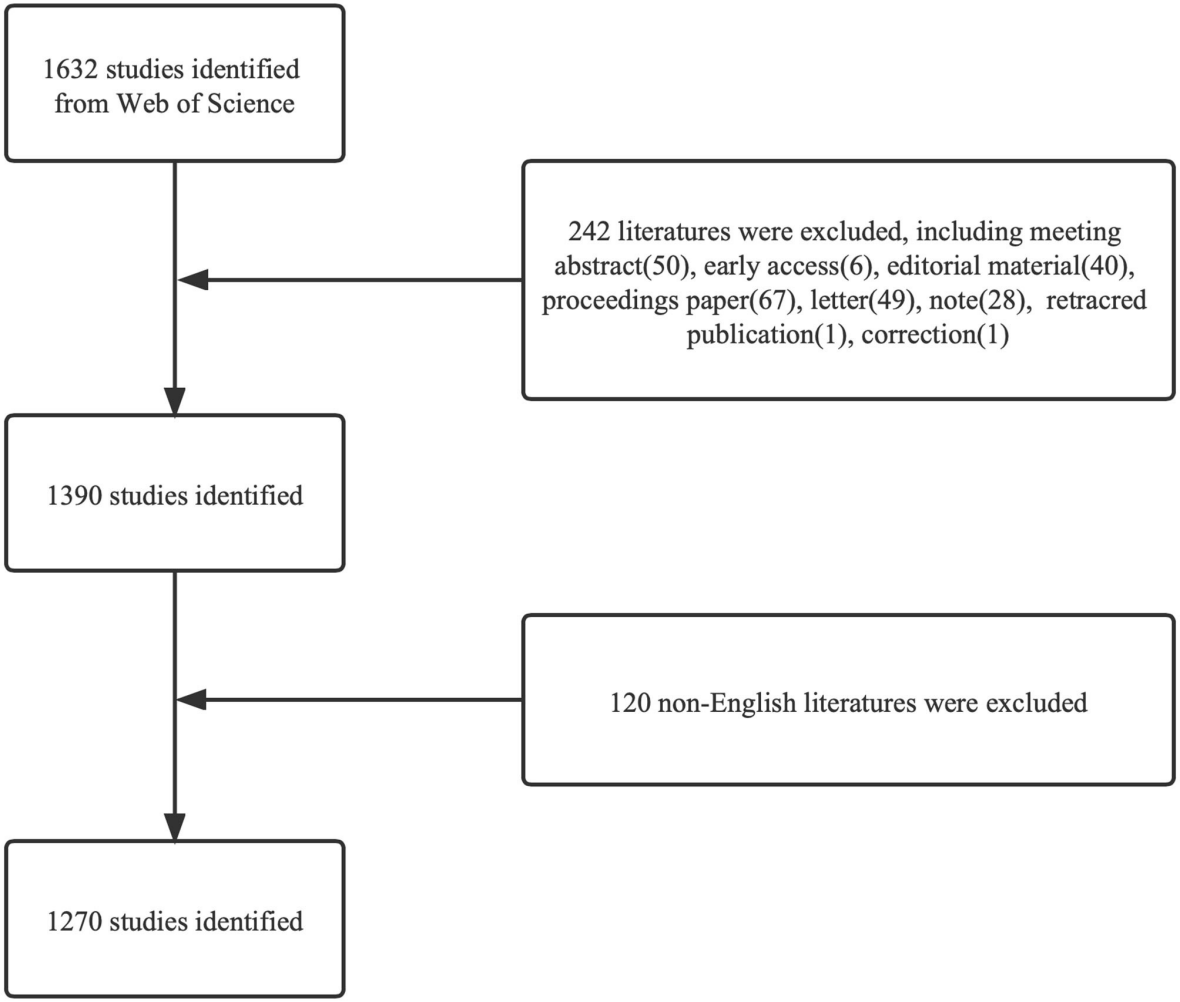
Flowchart of the screening process.

The original data used in this study were downloaded from the SCI-expanded database in the Web of Science Core Collection on 25 June 2021. The search was finished on the same day to eliminate any bias caused by the database update. The search strategies used in this study were as follows: [TS = (bronchoscopies OR bronchoscopic OR bronchoscopy OR tracheoscopic OR tracheoscope OR tracheobronchoscope) AND TS = (anesthesia OR anesthesia OR anesthesi^*^ OR anaesthesi^*^)]. Only original articles and reviews written in English were included, and the detailed screening process is shown in Figure 1.

### Data Collection

Raw data were initially extracted from the Web of Science SCI-expanded database. Two authors (KM and YW) independently searched information online and set the primary database, including the number of papers, titles, keywords, journals, institutions, Hirsch index (H-index), countries/regions, and citations, and reached a consensus after making comparisons. Although inaccurate analysis might not be avoided completely due to multiple versions of cited references and different forms of cited journals, we believe that most original data are reliable. Before data analysis by VOSviewer v.1.6.11, a thesaurus file was used for merging some duplicates into one word, correcting the misspelled elements, and deleting irrelevant words. Finally, the cleansed data were used for bibliometric analysis.

### Statistical Analysis

Microsoft Excel 2016 was used to analyze the total number of publications (Np), citation frequency, the number of citations without self-citations (Nc), countries, institutes, journal sources, H-index, and impact factor (IF). The H-index is a mixed quantitative indicator used to evaluate the academic productivity and the academic contribution of a researcher, and it could also be used to describe the publication output of an institution, a nation, or a journal (11, 12). The IF is an important indicator that measures the quality and impact of medical journals (13).

Furthermore, literature keywords, co-cited frequencies, and research hotspots were evaluated in the network analysis. VOSviewer (1.6.11) was used to identify productive countries/regions, institutions, and keywords, and to construct related visual networks. In the VOSviewer network maps, different nodes indicate components, such as countries/regions and institutions (14). The size of the nodes reflects the number of studies or co-occurrence frequencies. The larger the node, the more times the term appears. Links between nodes represent the co-occurrence relationships, and the size of the links indicates the co-occurrence frequencies of the two nodes. The link strength between nodes reflects the co-occurrence frequency of terms. The total link strength is the sum of the link strengths of a term relative to all other terms. CiteSpace (5.7.R5W), a software tool for creating visual maps and exploring maps based on bibliographic data (15), was used to detect the references with strong citation burstness to identify emerging topics. R (Version 4.0.2) is the language and environment for statistical computing and graphics. The Bibliometrix package in R was used to further illustrate the changes in the annual document (16).

## Results

### Overview of Publications on Bronchoscopy Anesthesia

A total of 1,270 publications were extracted from Web of Science. Based on the inclusion criteria, all the publications related to tracheoscopy anesthesia were extracted from Web of Science. The total number of citations was 25,504 (23,136 without self-citation), and the average citing frequency was 20.08 times per article. The H-index of all the publications related to bronchoscopy anesthesia was 70.

### The Annual Trends of Bronchoscopy Anesthesia-Related Publications

**Figure 2:**
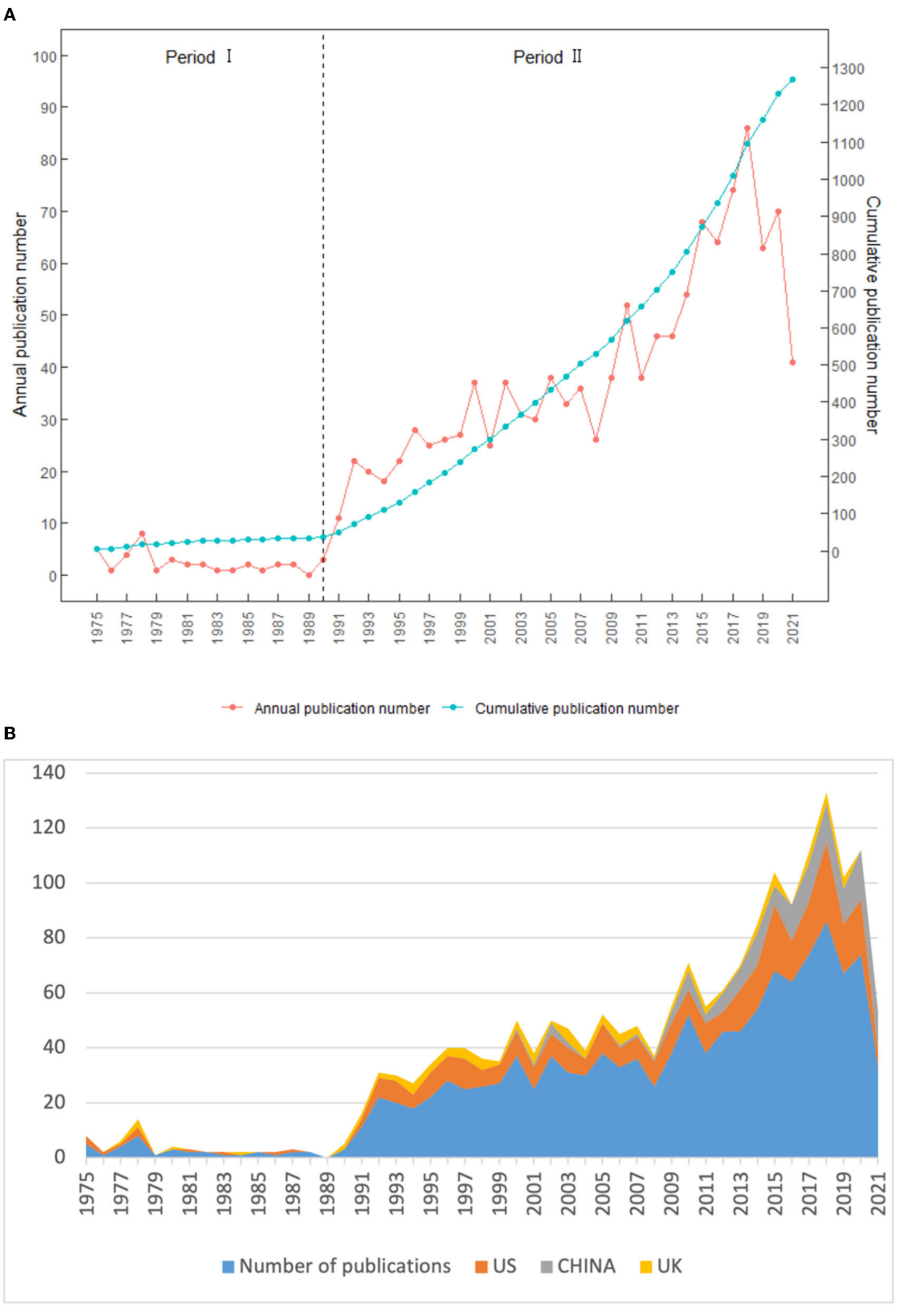
**(A)** Number of publications by year. **(B)** Number of publications by year of US, China, and UK.

The annual trends of the publications related to bronchoscopy anesthesia was shown in Figure 2A. It was found that the first article in this field appeared in 1975. From then on, the years could be divided into two periods using the exponential growth model: period one, covering 15 years between 1975 and 1990, and period two, from 1990 to the present. Period one was a rudimentary stage in the research on bronchoscopy anesthesia. The number of publications per year was less than ten, with a mean of 2.4, showing no significant increase because the theories concerning bronchoscopy anesthesia were not well developed at that time. In period two, the Np increased gradually, and especially since 2014, the number of articles issued each year exceeded 60 reaching the peak (75, 6%) in 2018 because of the rapid development of professional theories in this field during this period. In general, the Np increased from 5 in 1975 to 74 in 2020, demonstrating that bronchoscopy anesthesia had attracted increasing attention and interest among global researchers.

### Global Contribution to Publications on Bronchoscopy Anesthesia

**Figure 3:**
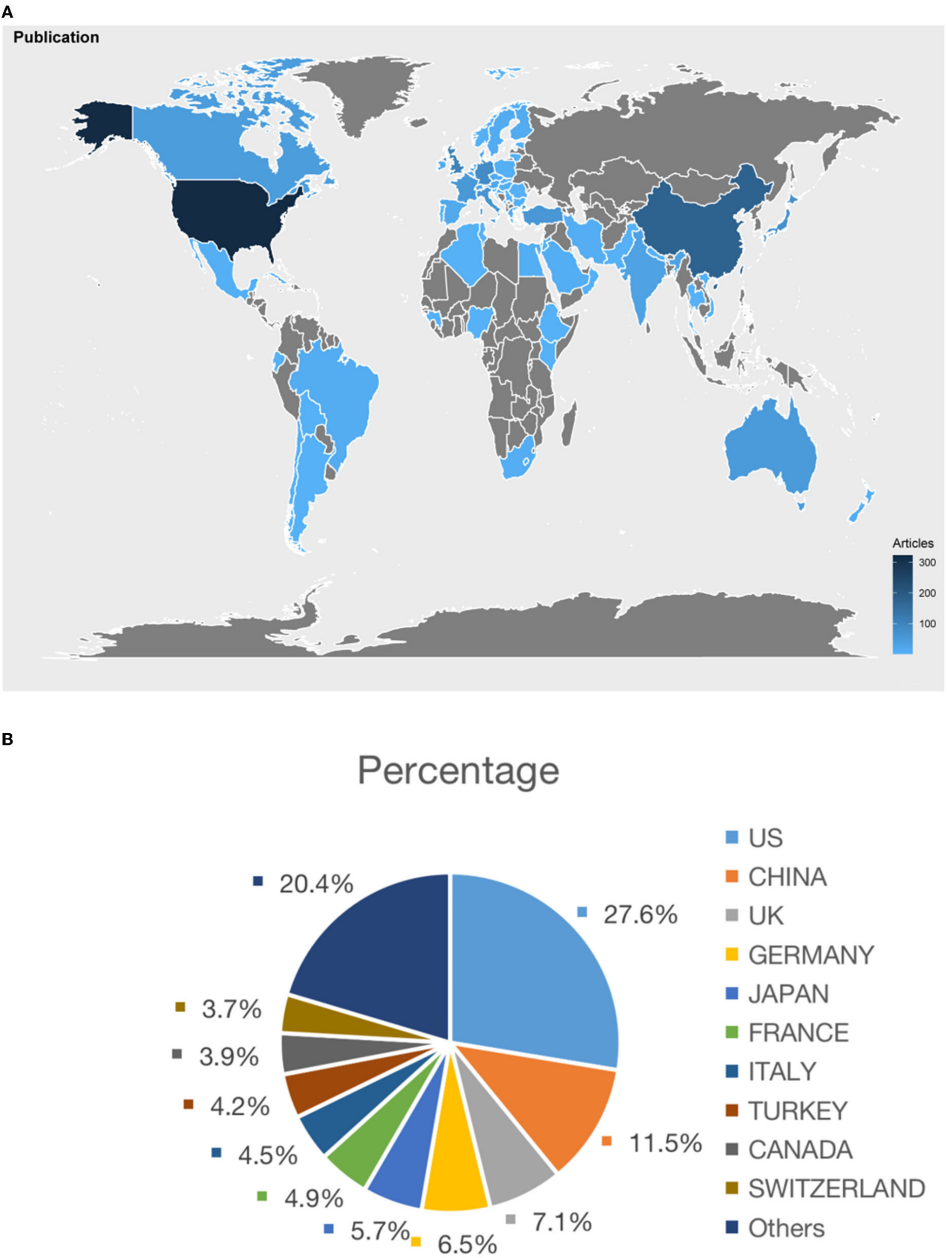
**(A)** The regional distribution. **(B)** Distributions and percentages of countries/regions of publications.

**Figure 4:**
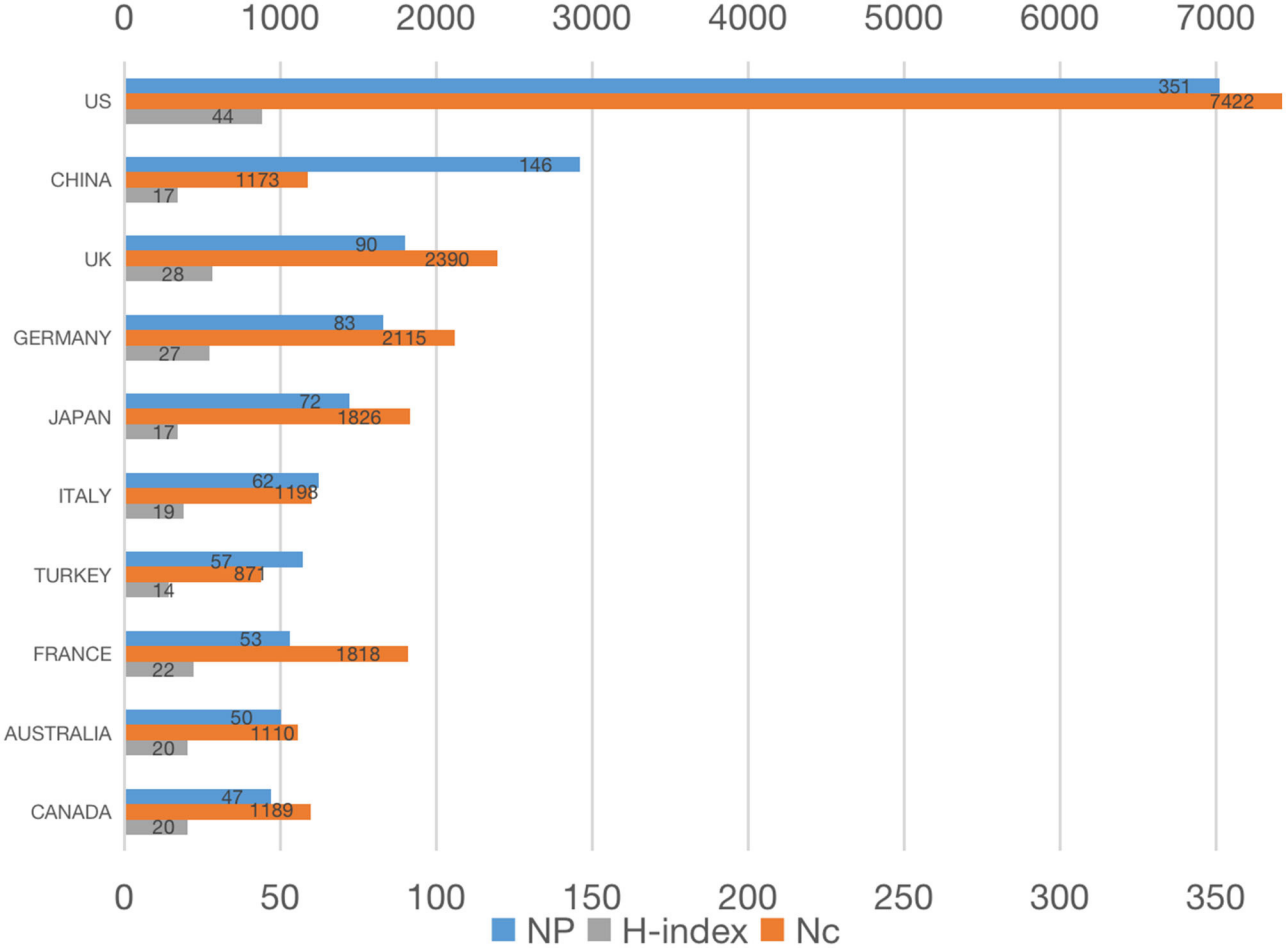
Total publications, H-index, and the total citation of top 10 countries/regions.

As shown in Figure 3A, the color block of the US, China, and Europe was darker than that of other countries and regions, representing greater contributions to the research of bronchoscopy anesthesia. Figure 3B showed the top 10 countries/regions of all publications in order of the number of publications. The US ranked first in the number of publications (351, 27.6%), followed by China (146, 11.5%) and the UK (90, 7.1%). According to the growing trend in different countries (Figure 2B), the US took the lead in the research of bronchoscopy anesthesia and contributed the most with a steady upward trend. It is worth noting that China began research in this field relatively late in 2000, but it has shown an apparent growing trend in this field since 2009. The growth trend in the UK was more fluctuating compared with the US and China. Figure 4 showed the top 10 countries with Np, Nc, and H-index, which were considered key measures designed to evaluate the quality of papers. The published literature in the US was cited 7,422 times, ranking first in all countries/regions, and achieved the highest value of H-index of 44 as well. The second most-cited country was the UK, with 2,390 citations and an H-index of 28. Germany ranked third with 2,115 citations and an H-index of 27.

### Institutions Publishing Papers on Bronchoscopy Anesthesia

**Figure 5:**
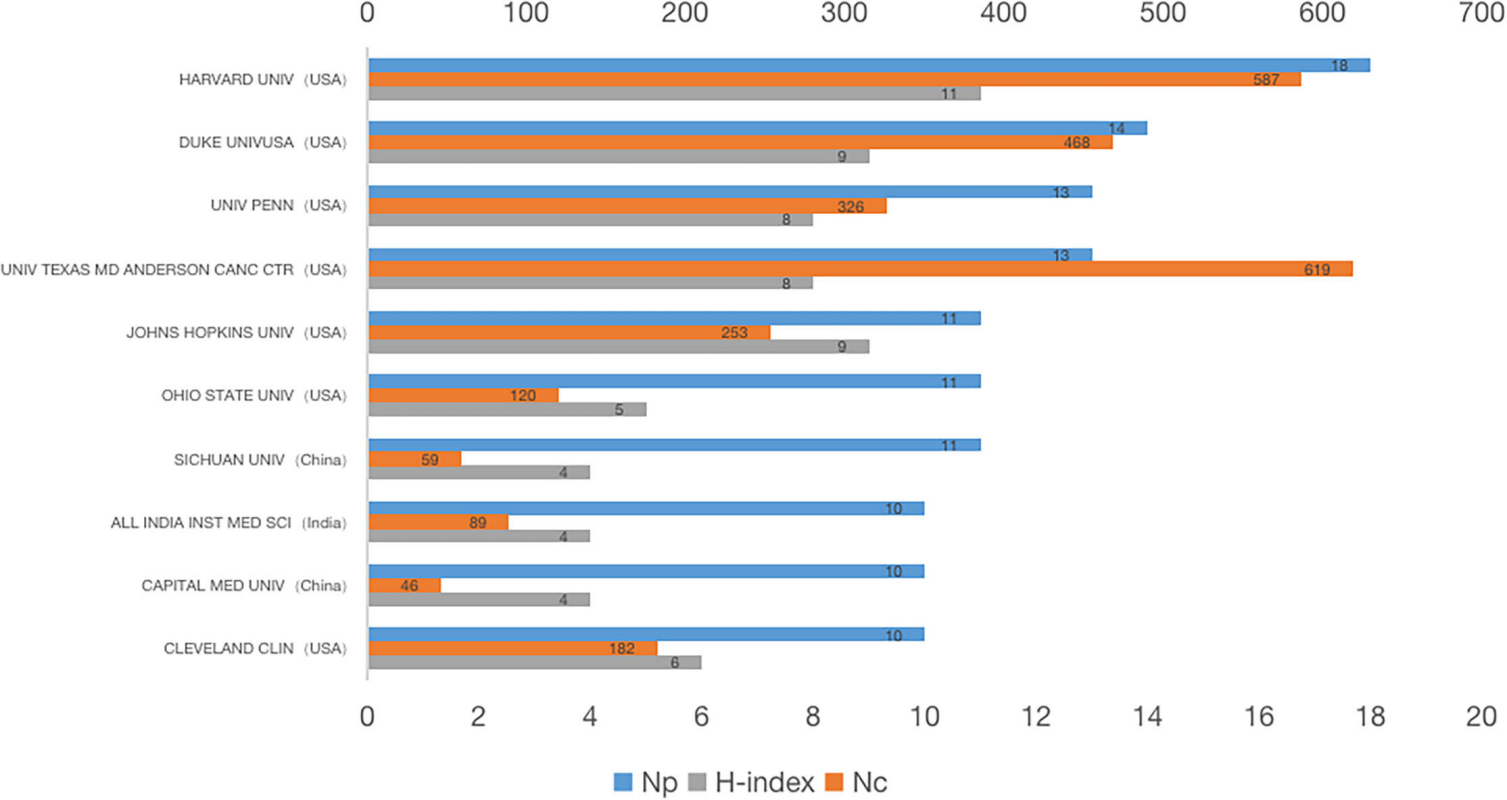
Total publications, H-index, and the total citation of top 10 institutes.

Figure 5 listed the top 10 institutions that published the most papers in this field. The most productive institution on bronchoscopy anesthesia was Harvard University (18), followed by Duke University (14) and the University of Pennsylvania (13). Citation frequency of papers from the University of Texas MD Anderson ranked first (619 citations), followed by Harvard University (587 citations) and Duke University (468 citations). In terms of the H-index, Harvard University ranked first (11), followed by Duke University (9) and Johns Hopkins University (9). Most of the top 10 institutions were from the US, excluding Sichuan University, the Capital Medical University of China, and the All India Institute of Medical Sciences of India. Although the number of publications from Chinese institutions was on the top 10 list, their H-index and Nc lagged behind those of other top 10 universities. Undoubtedly, the US took the lead in bronchoscopy anesthesia research.

### Journals Publishing Papers on Bronchoscopy Anesthesia

The top 10 journals contributing to bronchoscopy anesthesia were listed in Table 1. They accounted for 29.84% (379) of all papers on bronchoscopy anesthesia. *CHEST* (IF 9.41) ranked first with 72 publications, and *Anesthesia and Analgesia* (IF 5.108) with 46 articles came next. In addition, *Respiration* (IF 3.58), *Pediatric Anesthesia* (IF 2.556), and *International Journal of Pediatric Otorhinolaryngology* (IF 1.675) ranked third, fourth and fifth place, respectively. Among the top 10 journals, other than *Pediatric Anesthesia, International Journal of Pediatric Otorhinolaryngology*, and *Acta Anaesthesiologica Scandinavica*, the IF of other journals was higher than 3. According to *Journal Citation Reports, CHEST* and the *British Journal of Anaesthesia* were listed under Q1, the highest-ranked journals in a category.

**Table 1:** The top 10 most active journals.

**Rank**	**Journal**	**Np**	**H-index**	**Nc**	**IF (2020)**	**JCR**
1	Chest	72	35	3,717	9.41	Q1
2	Anesth Analg	43	22	1,646	5.108	Q2
3	Respiration	42	18	878	3.58	Q2
4	Pediatr Anesth	38	16	604	2.556	Q2
5	Int J Pediatr Otorhi	36	13	566	1.675	Q3
6	J Thorac Dis	34	10	225	2.895	Q3
7	Brit J Anaesth	32	20	913	9.166	Q1
8	Acta Anaesth Scand	29	13	464	2.105	Q3
9	Eur J Anaesth	28	12	332	4.33	Q3
10	Can J Anaesth	25	14	673	5.063	Q2

### Highly Cited Publications on Bronchoscopy Anesthesia

To analyze the most influential papers in this field, we shortlisted the top 10 publications with the most citations and listed them in Table 2 in terms of the title, first author, journal, publication year, and citation numbers. Two of the top 10 papers were published between 1991 and 2000, six between 2001 and 2010, and two between 2011 and 2017. As we mentioned above, the research on bronchoscopy anesthesia increased in period two from 1990 onward, and all the top 10 papers were published during this period, symbolizing the rapid progress in this field. The work of Yasufuku et al. ranked first with the highest Nc (478). In this paper, the authors evaluated the clinical efficacy of the newly developed ultrasound puncture bronchoscopy to visualize real-time transbronchial needle aspiration (TBNA) of the mediastinal and hilar lymph nodes under direct endobronchial ultrasonography (EBUS) guidance (17). The paper, “*Morbid obesity and tracheal intubation*”, ranked second. It concluded that obesity alone was not a predictive factor of difficult tracheal intubation (18). The paper written by Tanaka et al., comparing the clinical efficacy of surgical stabilization and internal pneumatic stabilization in severe flail chest patients after receiving prolonged ventilatory support, ranked third (19).

**Table 2:** The top 10 most active papers.

**Rank**	**Title**	**First Author**	**Year**	**Journal**	**Nc**	**IF (2020)**
1	Real-time endobronchial ultrasound-guided transbronchial needle aspiration of mediastinal and hilar lymph nodes.	Yasufuku K.	2004	Chest	478	9.41
2	Morbid obesity and tracheal intubation.	Brodsky J. B.	2002	Anesth Analg	296	5.108
3	Surgical stabilization of internal pneumatic stabilization? A prospective randomized study of management of severe flail chest patients.	Tanaka H.	2002	J Trauma	280	2.512
4	Exhaled nitric oxide correlates with airway eosinophils in childhood asthma.	Warke T. J.	2002	Thorax	210	7.892
5	The laryngeal mask airway.Its uses in anesthesiology	Pennant J. H.	1993	Anesthesiology	210	7.892
6	Thoracoscopy–state of the art.	Loddenkemper R.	1998	Eur Respir J	193	16.671
7	Complications,consequences,and practice patterns of endobronchial ultrasound-guided transbronchial needle aspiration:Results of the AQuIRE registry.	Eapen G. A.	2013	Chest	192	9.41
8	The incidence of aspiration associated with the laryngeal mask airway-a metaanalysis of published literature	Brimacombe J. R.	1995	J Clin Anesth	185	9.452
9	Diagnostic yield and complications of bronchoscopy for peripheral lung lesions results of the aquire registry	Ost David E	2016	Am J Resp Crit Care	181	21.405
10	Incidence of and risk factors for pulmonary complications after non-thoracic surgery	McAlister F. A.	2005	Am J Resp Crit Care	178	21.405

### Keywords Analysis

**Figure 6:**
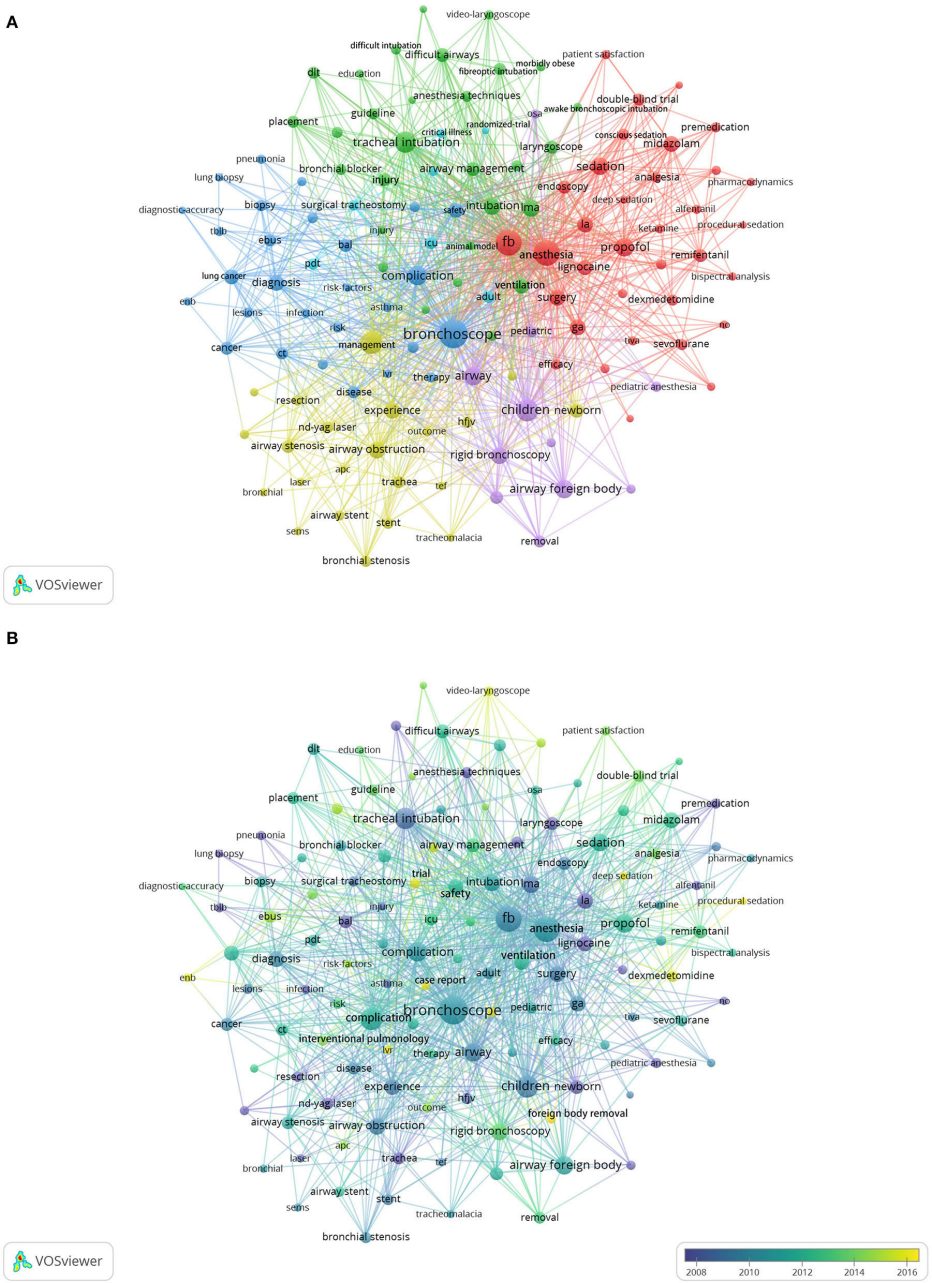
**(A)** Analysis of keywords. **(B)** Visualization of time when a keyword appears.

Considering the abundance of keywords, the minimum number of occurrences of keywords was set as 11. Of the 3,056 keywords, 131 were selected for co-occurrence analysis. As shown in Figure 6A, they were divided into six clusters. The first one, composed of 34 keywords (red circles), was mainly about applying sedatives and analgesics in bronchoscopy anesthesia. The second cluster was composed of 29 keywords (green circles) and mainly about difficult airway intubation and anesthesia management. The third cluster, consisting of 28 keywords (blue circles), was mainly about the application of bronchoscopy in the examination and treatment of various lung diseases. The fourth cluster of 22 keywords (yellow circles) concentrated on the treatment of airway stenosis and obstruction and intraoperative ventilation. The fifth cluster, with 11 keywords (purple circles), related to pediatric anesthesia during the removal of airway foreign bodies (AFBs) using rigid bronchoscopy. The sixth cluster was composed of 7 keywords (cyan circles) and focused on percutaneous dilatational tracheostomy with bronchoscopic guidance in the intensive care unit (ICU).

The top 10 keywords were listed in Table 3. The top frequent occurrences of keywords were “bronchoscopy,” “fiberoptic bronchoscopy,” “anesthesia,” and “management”, suggesting that studies related to bronchoscopy anesthesia mainly focused on airway management of various anesthesia methods in the process of bronchoscopy diagnosis and treatment. The colors of all keywords were divided by VOSviewer according to the year of publication. In Figure 6B, the keywords in purple appeared earlier than those in yellow. Yellow circles indicated the newest occurring keywords, including clinical trials of a combination of remifentanil and dexmedetomidine or other methods of sedation (20–22). For example, it was safer and more comfortable to use deep sedation when removing airway foreign bodies in children, indicating that the application of a combination of remifentanil and dexmedetomidine in painless bronchoscopy might be the priority and hotspot issue in future research on bronchoscopy anesthesia (23). Besides, electromagnetic navigation bronchoscope (ENB), video-laryngoscope, and lung volume reduction (LVR) were the newest occurring keywords.

**Table 3:** The top 10 most frequently used keywords.

**Rank**	**Keywords**	**Occurrences**
1	Bronchoscope	408
2	Fiberoptic bronchoscopy	306
3	Anesthesia	287
4	Management	199
5	Children	196
6	Tracheal intubation	163
7	Complication	135
8	Airway	121
9	Propofol	112
10	Airway foreign body	110

### References With Citation Burstness

**Figure 7:**
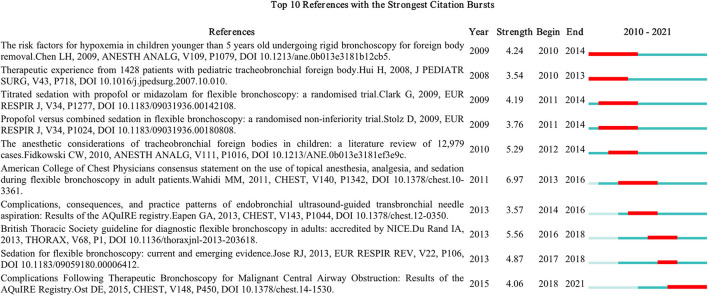
Top 10 references with strong citation burstness (MD = 2). The red bars mean some references are cited frequently; the blue bars represent references cited infrequently.

For further study of the related co-citation references, we conducted CiteSpace to investigate citation burstness. Citation burstness indicates references that attract more attention from scholars in a specific field during a particular period. Figure 7 illustrated the top 10 references with the strongest citation bursts. The minimum duration of the burst was set for 2 years, and a red line segment represented the initial and final years of the burst duration associated with academic circles for a specific period (24).

Among the top 10 references, the strongest burstness (*n* = 6.97) was caused by the paper, “*American College of Chest Physicians consensus statement on the use of topical anesthesia, analgesia, and sedation during flexible bronchoscopy in adult patients*,” authored by Wahidi et al. with citation burstness from 2013 to 2016 (25). This was followed by the paper, “*The anesthetic considerations of tracheobronchial foreign bodies in children: a literature review of 12,979 cases*,” published in 2010 by Fidkowski et al. (26). The research titled “*Complications Following Therapeutic Bronchoscopy for Malignant Central Airway Obstruction: Results of the AQuIRE Registry”* (27), also published in 2010, emerged third.

## Discussion

In this study, we undertook a bibliometric analysis to investigate the developmental trends and hotspots of research on bronchoscopy anesthesia using the data extracted from the SCI-expanded database using VOSviewer, CiteSpace, and Bibliometrix software. We retrieved a total of 1,270 original articles and reviews published since the establishment of the database. As noted in Figure 2A, there was an overall upward trend in the annual number of publications, especially after 1990. The growth rate peaked in 1991, and one of the main reasons for this was the application of local anesthetic lidocaine for fiberoptic bronchoscopy. This not only facilitated the diagnosis and treatment on the part of the doctor but also improved the comfort of the patient. Owing to the rapid development of professional theories in period two, more researchers focused on bronchoscopy anesthesia. An analysis of citation bursts is shown in Figure 7. These citation bursts mainly focused on 2009–2015, and the number of articles published worldwide also increased rapidly during this period (Figure 2A). We speculated that it was related to the issuance of authoritative guidelines. With the publication of “*The American College of Chest Physicians consensus statement on the use of topical anesthesia, analgesia, and sedation during flexible bronchoscopy in adult patients*,” in 2011 (25), all physicians performing bronchoscopy were asked to consider using topical anesthesia, analgesics, and sedative agents. Subsequently, the “*British Thoracic Society guideline for diagnostic flexible bronchoscopy in adults: accredited by NICE*” published in 2013 gave anesthesiologists and bronchoscopists authoritative guidance (28).

The publications from various countries were then selected from the total list of publications. Among the top 10 countries/regions, the US ranked first in Np (27.6%), demonstrating that the US was the most productive country in research on bronchoscopy anesthesia. Seven of the top 10 affiliations in the research on bronchoscopy anesthesia were from the US. The H-index and Nc of the US also ranked first among the top countries/regions, and one of the reasons for this was that the US scholars started the research in this field earlier. As a developed country, the US allocates a large budget to research, has a vast number of research centers, and the quality of publications from the US is more likely to be acclaimed internationally. Although China ranked second in Np, the H-index and Nc of the papers from China were not high enough when compared with the other countries/regions in the top 10 category. The reason for this phenomenon may be a late start by China in this field. To improve this situation, more conscientious efforts need to be made by Chinese scholars in this field. The rapid growth of high-quality literature from China over the last decade also indicates that the research quality in China has improved quickly.

When it comes to journals, high IF journals reflect the quality of articles. Remarkably, *CHEST* ranked first in terms of the number of publications, citations, and H-index, indicating that it had a huge impact on bronchoscopy anesthesia. Three of the top 10 papers were from *CHEST*, demonstrating that it occupied an important status in the field of bronchoscopy anesthesia. This reminded scholars interested in this field to pay more attention to this journal. Among the top ten articles, nine were published in high-IF journals, which meant that publishing research on bronchoscopy anesthesia in high-quality journals was possible.

Keywords can provide information about the core content of an article, and the most frequent and newest keywords can be used to identify the dynamics of research trends and hotspots in a particular domain during the study period. As seen in Table 3, the keywords “anesthesia,” “bronchoscope,” and “management” were the most common and showed the greatest increase over time. Furthermore, all the top keywords with the strongest citation burst were related to the application of fiberoptic bronchoscopy in the removal of foreign bodies in the airway of children, as well as airway management and related complications during the diagnosis and treatment using bronchoscopy. Comparing Figures 6A,B, dexmedetomidine applied to procedural sedation (cluster 1) has become a hotspot over the last 5 years. Dexmedetomidine was associated with fewer incidents of oxygen desaturation and a reduced need for oral cavity suction than remifentanil during flexible bronchoscopy (21). Compared with midazolam, pre-injection of dexmedetomidine before induction significantly decreased the incidence of dreaming in patients undergoing flexible bronchoscopy during general anesthesia; as most dreams were pleasant, patients rarely kept painful memories. The application of dexmedetomidine improved sedative effectiveness with fewer procedural interruptions, shorter time to ambulation, and higher bronchoscopist satisfaction (20, 22). Therefore, we need to pay more attention to precision anesthesia and comfort in the future.

With progress in society, medical technology has also developed rapidly. As seen in Figure 6B, video laryngoscopy, ENB, and LVR were research hotspots over the last 5 years. The invention of video laryngoscopy reduced the risk of difficult airways. Research showed that video laryngoscopy for awake tracheal intubation was associated with a shorter intubation time, thus increasing the intubation effectiveness. Its success rate and safety profile seemed comparable to those of fiberoptic bronchoscopy (29, 30). In addition, ENB was used to locate small peripheral pulmonary nodules and guide surgical resection safely and accurately. ENB is safe and feasible with a high diagnostic success rate in interventional pulmonology in low resource settings under moderate sedation, which is beneficial to early detection and early treatment of small lung lesions (31, 32). Bronchoscopic lung volume reduction (BLVR) coil treatment is an alternative and promising treatment modality for selected severe emphysema patients (33). Therefore, improvements in surgical methods and advancements in surgical instruments have made it possible to treat intractable diseases.

Among the top productive 10 articles (Table 2), five articles addressed the progress in bronchoscopic examination, diagnosis, and treatment methods, and four articles highlighted airway assessment and management of anesthesia during the operation. In general, the advancement in surgical equipment and methods is inseparable from appropriate anesthesia. The synergy between improvements in medical equipment and precise anesthesia methods will become the focus of future research.

## Limitations

To the best of our knowledge, this is the first bibliometric analysis of hotspots and dynamic pilot study on bronchoscopy anesthesia over the past decade. In addition, VOSviewer, Citespace, and Bibliometrix were applied to perform the survey simultaneously, enabling our research results to be more accurate and objective. Nevertheless, the present research has some limitations. First, we only chose Web of Science Core Collection and ignored other search engines such as PubMed and Scopus, knowing that different databases may produce different numbers of publications and citation counts. Second, only English articles were included, which may have decreased the number of retrieved articles. Finally, as our research was temporary and the count of relevant articles would also change as time goes on, the findings of this study are valid as of June 25, 2021. With the rapid updating of hot topics and research frontiers in bronchoscopy anesthesia, we might have missed some research hotspots.

## Conclusion

In this study, we discuss the developmental trends and research hotspots in bronchoscopy anesthesia and find that there has been a surge of interest in this field in recent decades. The US dominates this field, represented by the largest number of publications (351), the highest H-index (44), and extensive international collaborations. The selection and dosage of sedatives have always been the focus of disease research for the sake of improving comfort and safety. The clinical application of ENB has increased the diagnosis rate of minimal changes, and the diagnosis rate of the early lung tissue has increased. The invention of video laryngoscopy has reduced the risk of difficult airways, which not only shortens the intubation time but also increases the success rate. Medical development requires technological progress. In summary, our results revealed a comprehensive scientometric analysis of research on bronchoscopy anesthesia from a global perspective and may provide useful clues for future research directions and scientific decision-making in this domain.

## Data Availability Statement

The raw data supporting the conclusions of this article will be made available by the authors, without undue reservation.

## Author Contributions

KM, YW, and SW did this bibliometrics analysis and wrote manuscript. HY, HD, JW, WZ, HZ, XZ, and YG participated in experimental design and manuscript writing. XS and XL designed this study and organized the manuscript writing. All authors read and approved the final manuscript.

## Funding

This study was supported by grants from the Clinical Research Plan of SHDC (SHDC2020CR3044B) and the National Natural Science Foundation of China (82000085).

## Conflict of Interest

The authors declare that the research was conducted in the absence of any commercial or financial relationships that could be construed as a potential conflict of interest.

## Publisher's Note

All claims expressed in this article are solely those of the authors and do not necessarily represent those of their affiliated organizations, or those of the publisher, the editors and the reviewers. Any product that may be evaluated in this article, or claim that may be made by its manufacturer, is not guaranteed or endorsed by the publisher.

## Abbreviations

FB: fiberoptic bronchoscopy
DLT: double-lumen tube
EBUS: endobronchial ultrasound
COPD: chronic obstructive pulmonary disease
LVR: lung volume reduction
HFJV: high-frequency jet ventilation
LA: local anesthesia
TEF: tracheoesophageal fistula
NO: nitrous oxide
LMA: larynx mask airway
GA: general anesthesia
VBN: virtual bronchial navigation
ECMO: extracorporeal membrane oxygenation
APC: argon plasma coagulation
ENB: electromagnetic navigation bronchoscopy
OSA: obstructive sleep apnea
SEMS: self-expandable metallic stent
OSA: obstructive sleep apnea
CT: computed tomography
BAL: bronchoalveolar lavage
TBNA: transbronchial needle aspiration
PDT: percutaneous dilatational tracheostomy
TIVA: total intravenous anesthesia
TBLB: transbronchial lung biopsy.

## References

[1] Kamran A Zendejas B Jennings RW. Current concepts in tracheobronchomalacia: diagnosis and treatment. Semin Pediatr Surg. (2021) 30:151062. 10.1016/j.sempedsurg.2021.151062 34172207

[2] Blach J Frak M Krawczyk P Pankowski J Pankowski A Buczkowski J. Observational cross-sectional study of 5279 bronchoscopy results for the practical effectiveness of various biopsy techniques in the diagnosis of lung diseases with particular emphasis on lung cancer. BMJ Open. (2021) 11:e043820. 10.1136/bmjopen-2020-043820 34373288 PMC8354294

[3] Giri M Puri A Wang T Huang G Guo S. Virtual bronchoscopic navigation versus non-virtual bronchoscopic navigation assisted bronchoscopy for the diagnosis of peripheral pulmonary lesions: a systematic review and meta-analysis. Ther Adv Respir Dis. (2021) 15:17534666211017048 10.1177/17534666211017048 34057861 PMC8172954

[4] Haga T Cho K Nakagawa A Takagiwa J Arakawa S Sakamoto Y. Complications of fiberoptic bronchoscopy in very elderly adults. J Am Geriatr Soc. (2016) 64:676–7. 10.1111/jgs.13999 27000358

[5] Zhang K Xiao TK Chen ZQ Xiong SG Wang XH. Comparison of oxycodone hydrochloride and sufentanil used in painless fiberbronchoscopy. Eur J Inflamm. (2018) 16:5. 10.1177/2058739218774202

[6] Chen CM. Science mapping: a systematic review of the literature. Review. J Data Info Sci. (2017) 2:1–40. 10.1515/jdis-2017-0006

[7] Cancino CA Merigo JM Coronado F Dessouky Y Dessouky M. Forty years of computers & industrial engineering: a bibliometric analysis. Comput Indust Eng. (2017) 113:614–29. 10.1016/j.cie.2017.08.033

[8] Lee IS Lee H Chen YH Chae Y. Bibliometric analysis of research assessing the use of acupuncture for pain treatment over the past 20 years. J Pain Res. (2020) 13:367–76. 10.2147/JPR.S235047 32104058 PMC7023857

[9] Huang T Wu H Yang S Su B Tang K Quan Z. Global trends of researches on sacral fracture surgery: a bibliometric study based on VOSviewer. Spine. (2020) 45:E721–8. 10.1097/BRS.0000000000003381 31972744

[10] Ke LX Lu CC Shen R Lu TT Ma B Hua YP. Knowledge mapping of drug-induced liver injury: a scientometric investigation (2010-2019). Front Pharmacol. (2020) 11:842. 10.3389/fphar.2020.00842 32581801 PMC7291871

[11] Hirsch JE. Does the h index have predictive power? Proc Natl Acad Sci USA. (2007) 104:19193–8. 10.1073/pnas.0707962104 18040045 PMC2148266

[12] Jones T Huggett S Kamalski J. Finding a way through the scientific literature: indexes and measures. World Neurosurg. (2011) 76:36–38. 10.1016/j.wneu.2011.01.015 21839937

[13] Roldan-Valadez E Yoselin Salazar-Ruiz S Ibarra-Contreras R Rios C. Current concepts on bibliometrics: a brief review about impact factor, Eigenfactor score, CiteScore, SCImago Journal Rank, Source-Normalised Impact per Paper, H-index, and alternative metrics. Irish J Med Sci. (2019) 188:939–51. 10.1007/s11845-018-1936-5 30511320

[14] van Eck NJ Waltman L. Software survey: VOSviewer, a computer program for bibliometric mapping. Scientometrics. (2010) 84:523–38. 10.1007/s11192-009-0146-3 20585380 PMC2883932

[15] Chen C Chen Y. Searching for clinical evidence in CiteSpace. AMIA Annu Symp Proc AMIA Symp. (2005) 2005:121–5. PMC1560638 16779014

[16] Aria M Cuccurullo C. bibliometrix: an R-tool for comprehensive science mapping analysis. J Inform. (2017) 11:959–75. 10.1016/j.joi.2017.08.00733219880

[17] Yasufuku K Chiyo M Sekine Y Chhajed PN Shibuya K Iizasa T. Real-time endobronchial ultrasound-guided transbronchial needle aspiration of mediastinal and hilar lymph nodes. Chest. (2004) 126:122–8. 10.1378/chest.126.1.122 15249452

[18] Brodsky J Lemmens H Brock-Utne J Vierra M Saidman L. Morbid obesity and tracheal intubation. Anesth Anal. (2002) 94:732–6. 10.1097/00000539-200203000-00047 11867407

[19] Tanaka H Yukioka T Yamaguti Y Shimizu S Goto H Matsuda H. Surgical stabilization of internal pneumatic stabilization? A prospective randomized study of management of severe flail chest patients. J Trauma. (2002) 52:727–32. 10.1097/00005373-200204000-00020 11956391

[20] Chen L Zhang J He WS Liu W. Comparative effects of dexmedetomidine and midazolam on dreaming of patients undergoing flexible bronchoscopy during general anesthesia. Med Sci Monitor. (2021) 27:e929000. 10.12659/MSM.929000 33526763 PMC7866489

[21] Paul M Rastogi A Chatterje A Agarwal A Mishra P Khan A. Comparative evaluation of propofol and combination of propofol-dexmedetomidine in adjunct with topical airway anesthesia for rigid bronchoscopy: a randomized double-blinded prospective study. Ann Card Anaesth. (2021) 24:49–55. 10.4103/aca.ACA_45_19 33938832 PMC8081131

[22] Wu SH Lu DV Hsu CD Lu IC. The effectiveness of low-dose dexmedetomidine infusion in sedative flexible bronchoscopy: a retrospective analysis. Med Lith. (2020) 56:193. 10.3390/medicina56040193 32340204 PMC7231242

[23] Bi YM Ma YS Ni J Wu L. Efficacy of premedication with intranasal dexmedetomidine for removal of inhaled foreign bodies in children by flexible fiberoptic bronchoscopy: a randomized, double-blind, placebo-controlled clinical trial. BMC Anesthesiol. (2019) 19:10. 219. 10.1186/s12871-019-0892-6 31791239 PMC6886218

[24] Huang XQ Fan XWW Ying J Chen SY. Emerging trends and research foci in gastrointestinal microbiome. J Transl Med. (2019) 17:11. 67. 10.1186/s12967-019-1810-x 30819194 PMC6396506

[25] Wahidi MM Jain P Jantz M Lee P Mackensen GB Barbour SY. American College of Chest Physicians Consensus statement on the use of topical anesthesia, analgesia, and sedation during flexible bronchoscopy in adult patients. Chest. (2011) 140:1342–1350. 10.1378/chest.10-3361 22045879

[26] Fidkowski CW Zheng H Firth PG. The anesthetic considerations of tracheobronchial foreign bodies in children: a literature review of 12,979 cases. Anesth Anal. (2010) 111:1016–25. 10.1213/ANE.0b013e3181ef3e9c 20802055

[27] Ost DE Ernst A Grosu HB Lei X Diaz-Mendoza J Slade M. Complications following therapeutic bronchoscopy for malignant central airway obstruction results of the AQuIRE registry. Chest. (2015) 148:450–71. 10.1378/chest.14-1530 25741903 PMC4524328

[28] Du Rand IA Blaikley J Booton R Chaudhuri N Gupta V Khalid S. British Thoracic Society guideline for diagnostic flexible bronchoscopy in adults: accredited by NICE. Thorax. (2013) 68:1–44. 10.1136/thoraxjnl-2013-203629 23860341

[29] Smereka J Madziala M Dunder D Makomaska-Szaroszyk E Szarpak L. Comparison of Miller laryngoscope and UEScope videolaryngoscope for endotracheal intubation in four pediatric airway scenarios: a randomized, crossover simulation trial. Eur J Pediatr. (2019) 178:937–945. 10.1007/s00431-019-03375-y 30976922 PMC6511341

[30] Alhomary M Ramadan E Curran E Walsh SR. Videolaryngoscopy vs. fibreoptic bronchoscopy for awake tracheal intubation: a systematic review and meta-analysis. Review. Anaesthesia. (2018) 73:1151–1161. 10.1111/anae.14299 29687891

[31] Cherian SV Kaur S Karanth S Xian JZ Estrada-Y-Martin RM. Diagnostic yield of electromagnetic navigational bronchoscopy: a safety net community-based hospital experience in the United States. Ann Thorac Med. (2021) 16:102–9. 10.4103/atm.ATM_388_20 33680130 PMC7908899

[32] Wang LL He BF Cui JH Gao XL Chen PP Zhong WZ. Electromagnetic navigational bronchoscopy-directed dye marking for locating pulmonary nodules. Postgrad Med J. (2020) 96:674–9. 10.1136/postgradmedj-2019-137083 32041826

[33] Gulsen A. Importance of bronchoscopic lung volume reduction coil therapy in potential candidates for lung transplantation. BioSci Trends. (2018) 12:395–402. 10.5582/bst.2018.01134 30158333

